# Race and Length of Time Pursuing Pregnancy Among Women Who Utilized Medical Help to Get Pregnant 

**Published:** 2019-09

**Authors:** Emily Olig, Shanalee Mountan, James R Beal, Abe E Sahmoun

**Affiliations:** 1 School of Medicine, University of North Dakota, Dakota, USA; 2 Department of Family and Community Medicine, School of Medicine, University of North Dakota, Dakota, USA; 3 Department of Internal Medicine, School of Medicine, University of North Dakota, Dakota, USA

**Keywords:** Pregnancy, Infertility, Length of Time Pursuing Pregnancy, Race, Medical Help

## Abstract

**Objective:** The evaluation of racial disparities in access to and use of infertility services in the U.S. has been documented. The aims of this study were to: 1) investigate racial differences in length of time women report attempting to become pregnant until seeking medical help; and 2) determine the predictors of seeking medical help to achieve pregnancy.

**Materials and methods:** The National Survey of Family Growth 2011-2015 was used to analyze the duration women attempted to get pregnant among those who sought medical help.

**Results:** 563 women reported seeking medical help to achieve pregnancy. The majority 422 (81%) were white. Multiple linear regression showed that age (β = .93; p = .00), having less than high school education (β = 14.64; p = .01), and higher body mass index (β = .59; p = .00) are significantly associated with an increased length of time for seeking medical help to get pregnant. Religions other than Catholic or Protestant (β = -8.63; p = .04) is significantly associated with a decreased length of time for seeking medical help to get pregnant. Race was not associated with a significant difference in the length of time attempting to become pregnant (β = -1.80; p = .44).

**Conclusion:** Age, education attainment, religious affiliation, and body mass index are significantly associated with the length of time pursuing pregnancy. Once women have utilized medical resources, racial differences in the length of time pursuing pregnancy are not apparent.

## Introduction

Infertility is generally defined as failure to conceive after twelve or more months of unprotected sexual intercourse (1). The estimated prevalence of infertility varies significantly ranging from 3.5% to 24.1% depending on the infertility definition used, the timeframe, the method of assessment, country, and the study population (2, 3, 4). The measure of time to pregnancy has been used to assess biologic fertility (5). This approach has been extensively studied and its validity has been established (6). Racial differences in fecundity exist. For example, Thai primigravid women had a shorter time to pregnancy than European women (7). In the US, the National Surveys of Family Growth reported higher rates of infertility among Black (20%) and Hispanic (18%) than White women (7%) (8). While other studies found similar rates among Black women (7.2%), Hispanics (6.1%), Asians (5.6%), and Whites (5.5%) (9, 10). 

Minority groups often delay seeking infertility care compared to Whites (11, 12, 13, 14, 15, 16). For example, Black women are less likely than Whites to ever visit a doctor for help getting pregnant, and wait twice as long before seeking help when they do (9). Asian and Hispanic women also wait significantly longer prior to seeking fertility evaluation compared to White women (11, 17). Recent data revealed that the prevalence of using any medical service to help get pregnant was 40% lower for American-Indian/Alaska-Native and 47% lower for Black compared to White women and in 43% lower for Hispanic compared to non-Hispanic women (18). Chin et al. (9) showed that Black women were 46% less likely to seek help getting pregnant compared to White women. Even in a state with mandated and comprehensive insurance coverage for fertility services, disparities in access to these services exist, with the majority of women accessing those services are White, highly educated, and wealthy (19). Among women who do seek medical help to become pregnant, studies have shown that Black women wait longer to seek help after difficulty achieving pregnancy compared to White women (12, 15). Non-Hispanic White women are almost twice as likely to ever receive medical services for infertility compared to Hispanic and non-Hispanic Black women (20).

Access to care is not the only predictor of infertility service use. Schmidt et al. (21) found that only 47% of women who could not become pregnant sought infertility treatment, even though access to treatment is without co-payment. This suggests that the availability of free treatment is helpful, but insufficient, for patients to utilize infertility services. While racial disparities in initial access to infertility services have been established (9, 18, 22), to our knowledge the role of race on the length of time pursuing pregnancy in women who have utilized medical help to get pregnant has not been examined. The aims of this study were to: 1) investigate racial differences in length of time women report attempting to become pregnant until seeking medical help; and 2) determine the predictors of seeking medical help to achieve pregnancy.

## Materials and methods

***Data sources*** *:* We conducted a retrospective cross-sectional analysis of females, aged 18-44, with self-reported use of medical help to get pregnant using the combined datasets collected by the National Survey of Family Growth (NSFG) 2011-2013 and 2013-2015. The NSFG is a nationally representative survey conducted by the CDC’s National Center for Health Statistics, providing population-based estimates of infertility evaluation use. The 2011-2013 public use data files include 5,601 interviews with women 15-44 years of age living in households in the U.S. and the 2013-2015 public use data files include 5,699 interviews with women of the same age range (23).

Inclusion criteria were: women, age 18-44, with self-reported use of medical help to get pregnant. Exclusion criteria were women less than 18 years of age (non-adults), and women who received medical help to prevent miscarriage. Women who received medical help only to prevent miscarriage were excluded because of dissimilarities in the cause, types of services dispensed, and reasons for seeking care compared to women who are unable to achieve pregnancy (24). Women were then asked what specific type of medical help they, or their partner, had utilized.

***Demographic and clinical variables:*** Data included: age, race, education attainment, total family income, marital status, insurance type, body mass index, metropolitan status, religious affiliation, parity, memory or decision making problems, infertility testing, type of infertility treatments received, the length of time the woman has been trying to conceive, and the number of visits in the last 12 months to help get pregnant. Participants were asked: “Have you or your husband ever been to a doctor or other medical care provider to talk about ways to help you become pregnant?” The next follow up question was: “Think about all of the medical help you or your partners have ever received to help you become pregnant. Which of these services did you or your partner have to help you become pregnant?” Infertility services include: advice, ovulation drugs, drugs or surgery for endometriosis, surgery for blocked tubes, surgery for uterine fibroids, artificial insemination, in vitro fertilization or other assisted reproduction. The self-reported length of time pursuing pregnancy was determined by asking “How long have you been trying to get pregnant?”. 

Female respondents were asked for their racial background. Respondents were categorized into the following groups: non-Hispanic White, Black, or Other (Asian; American-Indian/Alaska-Native; Native-Hawaiian or Other Pacific Islander). The minority group (n = 141) for this study is made up of Blacks (n = 76) and others (n = 65). 

***Statistical analyses*** *:* Exploratory data analysis was performed using summary statistics and bivariate comparisons. We used multiple linear regression to adjust for confounders. All significance tests were two-sided; a *p* < .05 was considered significant. SAS v.9.4 (SAS Institute, Cary, NC) was used to analyze the data taking into account the NSFG’s complex sample survey design. Sampling errors were assessed using the adequate survey procedure and the NSFG documentation, which takes into account the clustered nature of the sample.

## Results

563 female respondents reported seeking medical help to get pregnant. 141(19%) women were of a minority race (Table 1).

**Table 1:** Demographic and reproductive characteristics of women in the NSFG 2011-2015

**Variables **	**Minority n (%)**	**White n (%)**	**P value**
Ever used medical help to achieve pregnancy (n = 563)	141 (19)	422 (81)	
Age respondent (years)			0.667
18-35	83 (44)	203 (48)	
36-44	58 (56)	219 (52)	
Education attainment			0.898
Less than high school	11 (4)	26 (4)	
High school graduate	34 (25)	100 (22)	
Some college	31 (19)	87 (17)	
College degree or higher	65 (52)	209 (57)	
Total family income			0.111
< $50 K	88 (50)	169 (36)	
$50 K - < $100 K	25 (23)	158 (40)	
$100 K+	28 (27)	95 (24)	
Marital status			0.023
Married	81 (68)	316 (81)	
Divorced/widowed/separated	20 (12)	52 (8)	
Never married	24 (9)	14 (2)	
Cohabiting	16 (11)	40 (9)	
Current health insurance status			0.802
Private insurance	86 (74)	295 (76)	
Medicaid, CHIP, other state-sponsored plan	29 (14)	56 (11)	
Medicare, military health care, government health care	8 (4)	19 (4)	
Single-service plan, Indian health service, uninsured	18 (8)	52 (9)	
Body mass index (kg/m^2^)			0.646
Underweight (18-< 18.5)	-	6 (2)	
Normal (18.5-24.99)	26 (28)	133 (33)	
Overweight (25-29.99)	38 (20)	83 (20)	
Obese (30–39.99)	48 (33)	135 (29)	
Morbidly obese (40+)	11 (5)	35 (8)	
Reported memory or decision making problems	23 (13)	44 (9)	0.286
Metropolitan [MSA] status			0.016
Principal city MSA area	64 (42)	112 (22)	
Other MSA	62 (51)	239 (63)	
Not MSA	15 (8)	71 (15)	
Current religious affiliation			0.005
No religion	17 (18)	76 (14)	
Catholic	25 (15)	109 (29)	
Protestant	83 (46)	205 (51)	
Other religions	16 (21)	32 (6)	
Parity*			0.054
No children	56 (41)	122 (24)	
One child	34 (21)	110 (27)	
Two or more children	51 (38)	190 (49)	
Infertility testing			0.140
Respondent	32 (20)	73 (16)	
Husband/Partner	9 (13)	24 (5)	
Both	42 (32)	177 (42)	
No one	58 (35)	148 (37)	
Infertility treatment			0.092
Advice only	60 (38)	144 (35)	
Ovulation +/- advice	29 (20)	120 (32)	
Ovulation + insem +/- advice	10 (5)	30 (6)	
Invitro** +/- any treatment	7 (10)	27 (6)	
Any treatment with invasive procedures	12 (6)	43 (10)	
Any treatment without invasive procedures	8 (4)	24 (4)	
Not specified	15 (17)	34 (7)	
Number of Visits in the last 12 months to help get pregnant§			0.942
Median (range)	2 (1-40)	1.5 (1-50)	
Length of trying to become pregnant before first seeking medical help (months)			0.586
Median (range)	11.5 (1-120)	11.4 (1-240)	

* Number of respondent’s biological children (18 or younger) living in household;

** In vitro fertilization or other assisted reproduction; PID: Pelvic inflammatory disease; NSFG: National Survey of Fertility Growth;

§ n = 107 (married and cohabiting)

33 (6%) women reported being currently pregnant. Bivariate analysis revealed no significant differences by race in the percentage of female respondent’s age, education attainment, total family income, health insurance status, body mass index (BMI), infertility testing, infertility treatment received, the length of time trying to become pregnant before seeking medical help, and reporting memory or decision making problems (Table 1). White women were significantly more likely to report being married (p = 0.023), not live in the metropolitan area (p = 0.016), and religious affiliation (p = 0.005) (Table 1). Multiple linear regression showed that age (β = .93; p = 0.000), less than high school education (β = 14.64; p = 0.011), and higher BMI (β = .59; p = 0.000) are significantly associated with an increased length of time for seeking medical help to get pregnant (Table 2). Other religions than Catholic or Protestant (β = -8.63; p = 0.037) is significantly associated with a decreased length of time for seeking medical help to get pregnant.

Race was not associated with a significant difference in the length of time attempting to become pregnant (β = -1.80; p = 0.438). Secondary analysis was performed to compare the length of time for seeking medical help to get pregnant between nulliparous women, who may represent a less diversified group with regard to persistency of trying, and couples with one or more children and found no difference (β = 1.01; p = 0.754) (Data not shown)

## Discussion

We did not find a significant difference between Whites and minorities in the length of time pursuing pregnancy in those reporting use of medical help to get pregnant. The overall average duration of length of time pursuing pregnancy is 18.2 months which is comparable to Bunting et al. (38) among women who had sought medical advice is 19.14 months.

We found that higher body mass index is associated with a longer length of time pursuing pregnancy before seeking medical help to get pregnant. This finding is important considering that the risk of infertility has been shown to be three times higher in obese than in non-obese women, and several studies have shown that obese women need a longer time to achieve pregnancy (25, 26, 27). Furthermore, overweight and obesity are significantly associated adverse pregnancy outcomes and premature labor (28). The biological mechanisms underlying these associations involve the ovulatory dysfunction among obese women, as the increase in adipose tissue amplifies its endocrinologic effects on follicular maturation and ovulation (28) which in turn results in anovulatory cycles that are commonly seen in patients with infertility. Knowing this underlying cause of anovulation will lead to treatment which can result in pregnancy or more regular menstrual cycles. Kazemijaliseh et al. (29) reported a high proportion of ovulatory disorder (39.7%) among infertile couples. 

**Table 2:** Multiple linear regression of length of time pursuing pregnancy among women who have utilized medical help to get pregnant using the NSFG 2011-2015

**Variables **	**Unadjusted**			**Adjusted**		
	**β**	**SE**	**P** ** value**	**β**	**SE**	**P** ** value**
Respondent’s age (years)	0.758	0.19	0.000	0.930	0.19	0.000
Respondent’s race						
Minority	-1.455	2.66	0.586	-1.801	2.31	0.438
Education attainment						
Less than high school	16.293	4.93	0.001	14.644	5.64	0.011
High school graduate	6.159	2.86	0.034	3.297	2.70	0.225
Some college	3.717	3.14	0.240	2.272	3.37	0.501
Total family income						
< $25 K	6.562	3.30	0.049	4.690	4.57	0.307
$25 K - < $50 K	7.755	2.75	0.006	4.346	2.78	0.122
$50 K - < $100 K	4.402	2.71	0.108	5.812	2.88	0.050
Marital status						
Married	1.849	2.75	0.503	4.102	3.08	0.186
Cohabiting	-0.164	4.77	0.973	2.130	5.21	0.684
Health insurance status						
Private insurance	-4.669	2.88	0.108	-3.329	3.01	0.271
Body mass index (kg/m^2^)	0.656	0.15	0.000	0.591	0.16	0.000
Metropolitan [MSA] status						
MSA area	-1.399	3.95	0.726	-0.599	3.77	0.874
Current religious affiliation						
Other religions	-8.090	4.47	0.073	-8.630	4.08	0.037
Catholic	0.362	4.53	0.937	1.255	4.11	0.761
Protestant	-3.156	4.08	0.441	-4.078	3.66	0.267
Parity*						
No children	2.132	2.97	0.475	3.387	3.01	0.263
One child	-1.240	2.62	0.392	-1.263	2.68	0.546

* Number of respondent’s biological children (18 or younger) living in household

Interestingly, weight loss through physical activity has been shown to be one of the most effective approaches to improving fertility outcomes in obese women (30). It is noteworthy that the impact of weight reduction alone becomes an ineffective strategy as the woman gets older (31).

We found that increasing age is associated with a longer length of time pursuing pregnancy in those reporting use of medical help to get pregnant. This is surprising since older women more frequently recognize a fertility problem and seek care than younger women (32, 33). However, increasing age and high-risk pregnancy were also reported as significant deterrent for seeking in infertility treatment (34). White and highly educated women more often pursue pregnancy at older ages (35). Parous women may delay seeking help to become pregnant (36). Perceiving infertility as a stigmatized condition (37) or fearing the label infertile (38) may delay medical help seeking for older women. It conceivable that women who are obese or at advanced age, assume they already know the reason for the delay in pregnancy and the ability to conceive, so women don't expect to conceive as quickly as others. However, the significant delay in seeking help is observed mainly among those women with less than high school education. Lastly, Akhondi et al. (39) reported that most of the couples will get pregnant within two years of unprotected sexual intercourse and thus, need no treatment suggesting giving more time to younger women to become pregnant maybe acceptable.

Finally, the definition of infertility is “restrained” for older women and the majority of health professionals consider a couple to be infertile if they have failed to conceive after 6 months instead of 12 months if the woman is over the age of 35 years due to the substantial need for close attention (40). However, the "restrained" definition for older women could be more of a reflection of the providers' concern that they don't have the “luxury of time” when trying to assist older women.

We found that women who reported religions other than Catholic or Protestant, is associated with a shorter length of time pursuing pregnancy for seeking medical help to get pregnant. Kessler et al. (24) reported that religion had no impact on seeking an initial evaluation or getting treatment. Most religions assert the importance of parenthood which implies a positive correlation between religion and infertility treatments use. However, some religions object to the use of certain medical devices or treatments such as contraceptives, vaccinations, or blood transfusions (41, 42). Many Christian churches are opposed to assisted reproduction (43). For example, in 1956 the Pope famously declared that artificial fecundation to be immoral because it dissociates procreation from sexual normal function (43). 

We found that less than high school education is associated with a longer length of time pursuing pregnancy. Datta et al. (44) reported that women with lower levels of education were less likely to have sought medical help for infertility. Lower education was also associated with a decreased likelihood of fertility treatment even when self-payment is not required (21).

As has been previously reported (20), we found that advice, infertility testing and ovulation drugs are the most commonly used services because of lower cost and less invasiveness.

Use of self-reported data is subject to recall bias. Race was self-reported which may result in bias as has been mentioned elsewhere (17, 45, 46). Because of the small sample size for the minority group it was not possible to analyze Blacks and others races separately. Women whose families have been in the U.S. for many generations differ from women newly immigrated. We also do not know to what extent cultural differences may have played a role in the null finding. Cross-sectional data are a snapshot in time, therefore we are unable to distinguish if a woman used medical help to get pregnant for her current period of infertility in which she reported the length of time she has been trying to get pregnant, or a prior attempt. Although time to pregnancy applies to couples, data are collected from the woman only. Time to pregnancy estimation is prone to several biases as mentioned elsewhere (47). We did not adjust for the number of obstetrics and gynecologists per state. The strengths of this study include the use of data representative of the national population and the analysis of several variables. Our analysis showed no difference by race regarding reported memory problems reducing the risk for misclassification bias.

In summary, increasing age, less than high school education, and higher body mass index, are significantly associated with longer length of time pursuing pregnancy. Race was not associated with the self-reported length of time attempting to get pregnant among women who sought medical help to get pregnant. Because race affects access to infertility care, interventions should focus on approaches that could help alleviate the discrepancy in access to these services. Initiatives such as didactic online resources and timely conversation initiation at the primary physicians’ office may reduce the gap. Future research should focus on educating women, 35 years and older, on the importance of seeking medical help within the first six months of trying. In addition to seeking fertility treatments, heavy women, younger than 35 years old, should be encouraged to lose weight through physical activity programs and diet counseling to maximize chances of successful pregnancy.

## Conclusion

The findings and conclusions of this study are those of the authors only and do not reflect the official policies of the Centers for Disease Control and Prevention’s (CDC), National Center for Health Statistics (NCHS), or the US Department of Health and Human Services.
